# Better Ways to Improve Standards in Brain-Behavior Correlation Analysis

**DOI:** 10.3389/fnhum.2012.00200

**Published:** 2012-07-16

**Authors:** Dietrich Samuel Schwarzkopf, Benjamin De Haas, Geraint Rees

**Affiliations:** ^1^Wellcome Trust Centre for Neuroimaging at University College LondonLondon, UK; ^2^Institute of Cognitive Neuroscience, University College LondonLondon, UK

Rousselet and Pernet (2012) demonstrate that outliers can skew Pearson correlation. They claim that this leads to widespread statistical errors by selecting and re-analyzing a cohort of published studies. However, they report neither the study identities nor inclusion criteria for this survey, so their claim cannot be independently replicated. Moreover, because their selection criteria are based on the authors’ belief that a study used misleading statistics, their study represents an example of “double dipping” (Kriegeskorte et al., 2009). The strong claims they make about the literature are therefore circular and unjustified by their data. Their purely statistical approach also does not consider the biological context of what observations constitute outliers.

In discussion, they propose that the *skipped correlation* (Wilcox, 2005) is an appropriate alternative to the Pearson correlation that is robust to outliers. However, this test lacks statistical power to detect true relationships (Figure 1A) and is highly prone to false positives (Figure 1B). These factors conspire to drastically reduce the sensitivity of this test in comparison to other procedures (Appendix 1). Further, it is susceptible to the parameters chosen for the minimum covariance estimator to identify outliers but these parameters are not reported.

**Figure 1:**
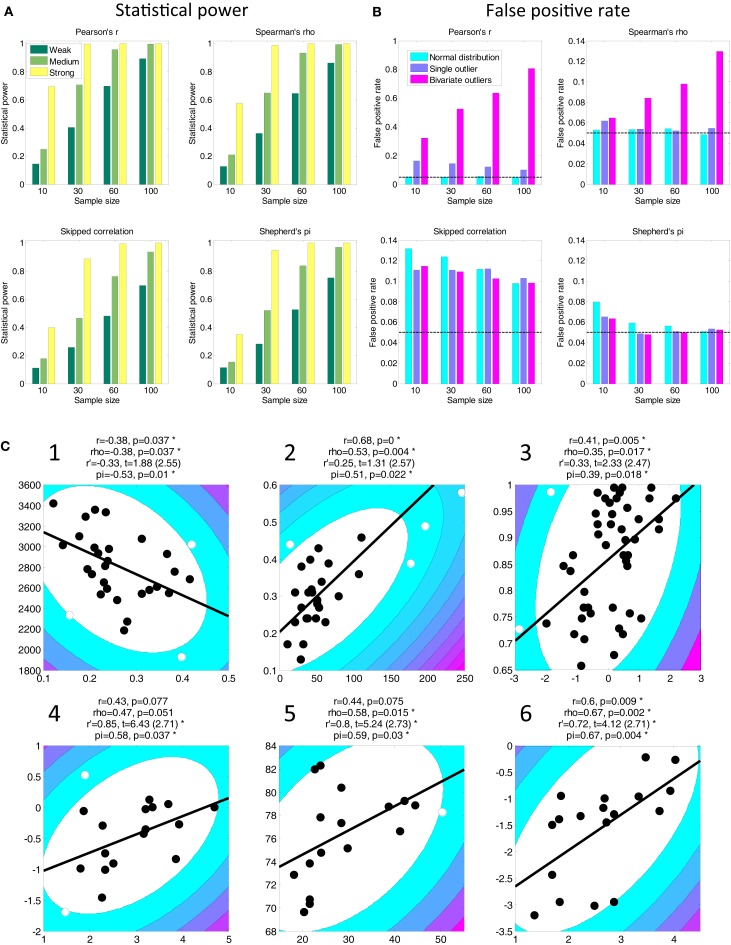
**Statistical power (A) and false positive rates (B) for four statistical tests and four sample sizes based on 10,000 simulations (see Appendix 1 for details)**. Outliers can drastically inflate false positives for Pearson correlation (note the difference in scale for this test). Skipped correlation (Wilcox, 2005) is generally very susceptible to false positives under all conditions. Only Shepherd’s *pi* provides adequate statistical power *and* protection against false positives. The black line in **(B)** denotes the nominal false positive rate of 0.05. **(C)** Replot of data shown in Rousselet and Pernet’s Figure 2. The contour lines indicate the bootstrapped Mahalanobis distance *D*_s_ from the bivariate mean in steps of six squared units (purple colors denote greater distances). Filled circles denote data included in the correlation, open circles denote outliers (see Appendix 2 for details). The solid line is a linear regression over the data after outlier removal. The correlation statistics shown are Spearman’s *rho*, skipped correlation *r*′ (critical *t* in parentheses), and Shepherd’s *pi*. Asterisks indicate significant results. All *p*-statistics rounded to third decimal. The freely available LIBRA toolbox (Verboven and Hubert, 2005) was used to calculate the skipped correlation. While the exact estimates of the *t*-statistic differ between R and MATLAB the conclusions about significance for these tests are very similar.

Their argument fails to consider a broad literature on robust statistics, although an extensive review is outside the scope of this commentary. We limit ourselves instead to presenting a practical alternative to their approach: Shepherd’s *pi* correlation (http://www.fil.ion.ucl.ac.uk/~sschwarz/Shepherd.zip). We identify outliers by bootstrapping the Mahalanobis distance, *D*_s_, of each observation from the bivariate mean and excluding all points whose average *D*_s_ is 6 or greater. Shepherd’s *pi* is Spearman’s *rho* but the *p*-statistic is doubled to account for outlier removal (Appendix 2). This compares very well in power (Figure 1A) to other tests and is more robust to the presence of influential outliers (Figure 1B). We replot the data Rousselet and Pernet presented in their Figure 2. The conclusions drawn from Shepherd’s *pi* are comparable to skipped correlation but less strict in situations where a relationship is likely (Figure 1C, Figures A1 and A2 in Appendix).

Consider for instance the data in Figure 1C-1. Pearson and Spearman correlation applied to these data are comparable. This implies that the assumptions of Pearson’s *r* were probably met in this case. The skipped correlation (*r*’) does not reach significance but nevertheless shows a similar relationship, consistent with our demonstration above that it is too conservative a measure. Under Shepherd’s *pi*, however, the relationship between these variables is significant. Indeed, reflecting our intimate knowledge of these data (Schwarzkopf et al., 2011), we already know that the relationship studied here replicates for separate behavioral measures (see Schwarzkopf et al., 2011 SOM). A similar pattern was observed for other data, e.g., Figure 1C-2. In some cases skipped correlation even removes the majority of data as outliers (e.g., their Figure 2E), which borders on the absurd.

Rousselet and Pernet also claim that none of the studies that they surveyed considered the correlation coefficient and its confidence intervals. Cohen defined that 0.3 < *r* < 0.5 constitutes correlations of medium strength (Cohen, 1988). Even “strong” correlations have *r* > 0.5, that is, at least 25% of the variance is explained. A correlation accounting for ~15% of variance is thus not particularly “modest” as they state. Naturally, this taxonomy is somewhat arbitrary but when relating complex cognitive functions to brain measures we are unlikely to find very high *r*, except for trivial relationships (Yarkoni, 2009).

Their failure to find reported confidence intervals in the literature is also puzzling because it does not accurately report the published work they considered. For example, our study, reproduced in their Figure 2A, reported bootstrapped 95% confidence intervals in the figure (Schwarzkopf et al., 2011). They also do not consider important aspects of what confidence intervals reflect. Naturally, a confidence interval *is* an indicator of the certainty with which the effect size can be estimated. However, it depends on three factors: the strength of the correlation, the sample size, and the data distribution. Because Pearson correlation assumes a Gaussian distribution we can predict the confidence interval for any given *r*. If the bootstrapped confidence interval differs from this prediction, the data probably do not meet the assumptions. Rousselet and Pernet’s example for bivariate outliers (their Figure 1D) illustrates this: the predicted confidence interval for *r* = 0.49 with *n* = 17 should be (0.01, 0.79). However, the bootstrapped confidence interval for this example is (−0.19, 0.87), much wider and also overlapping zero. This indicates that outliers skew the correlation and that it should not be considered significant. Compare this to Figure 1C-1 (their Figure 2A): the nominal confidence interval should be (−0.65, −0.02); the actual bootstrapped interval is very similar: (−0.67, −0.03). Therefore, the use of Pearson/Spearman correlation was justified here.

We propose simple guidelines to follow when testing correlations. First, use Spearman’s *rho* because it captures non-linear relationships. Second, bootstrap confidence intervals. Third, if the interval differs from the nominal interval, apply Shepherd’s *pi* as a more robust test. Fourth, estimate the reliability of individual observations, especially in cases where outliers strongly affect results. Outliers are frequently the result of artifacts or measurement error.

Our last point highlights an important general concern we have with Rousselet and Pernet’s argument. Statistical tests are important tools to be used by researchers for interpreting their data. However, the goal of neuroscience is to answer biologically relevant questions, not to produce statistically significant results. No statistical procedure can determine whether a biological question is valid or if a theory is sound. Rather, one has to inspect each finding and each data point in its own right, evaluating the data quality and the potential confounds on a case-by-case basis. Outliers should not be determined solely by statistical tests but must take into account biological interpretation (Bertolino, 2011; Schott and Düzel, 2011). And finally, there is only one way any finding can be considered truly significant; when upon repeated replication it passes the test of time.

## Figures and Tables

**Figure A1:**
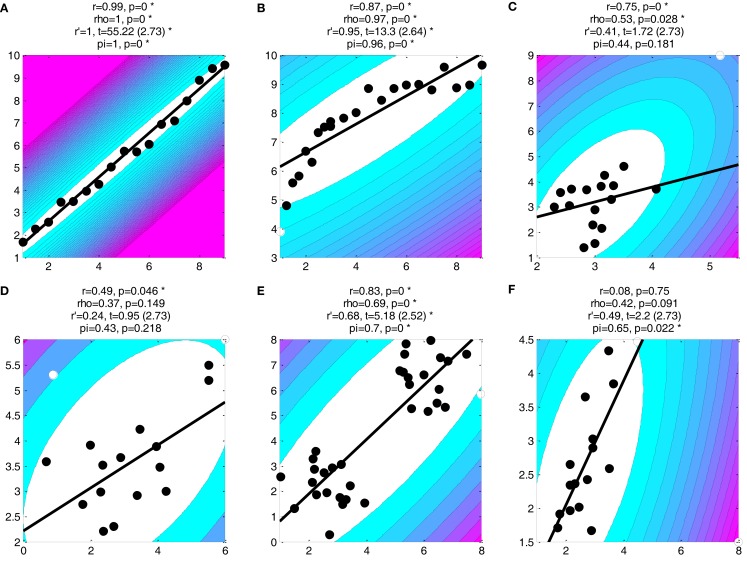
**Data shown in Figure 1 of Rousselet and Pernet’s article**. All conventions are as in Figure 1C.

**Figure A2:**
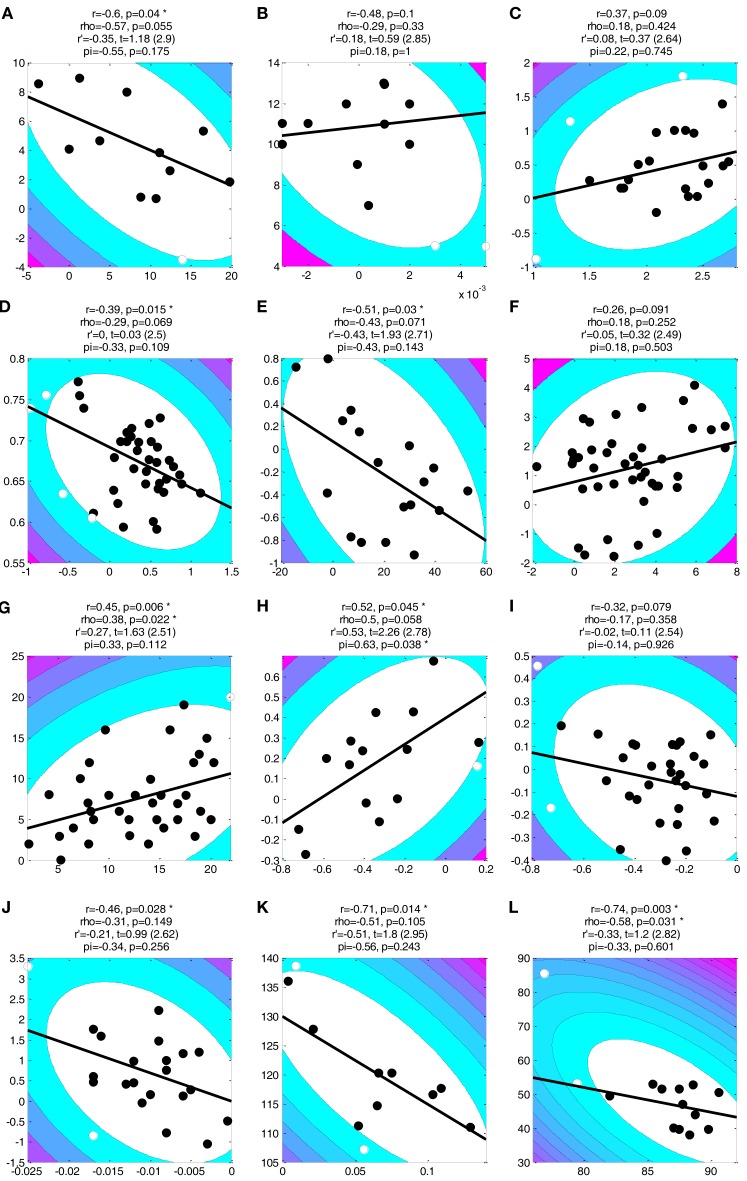
**Data shown in Figure A1 in Appendix of Rousselet and Pernet’s article**. All conventions are as in Figure 1C.

## Appendix 1

We ran a series of simulations to estimate statistical power and false alarm rates for a range of statistical tests and different conditions. All these analyses were performed in MATLAB 2010b (MathWorks, Inc.). The skipped correlation employs a minimum covariance estimator implemented in the LIBRA toolbox (Verboven and Hubert, 2005) for MATLAB (http://wis.kuleuven.be/stat/robust/LIBRA.html).

### Power simulations

To estimate the statistical power with which different tests can detect true correlations we examined a range of statistical tests on simulated data for which the ground truth is known. We simulated data for three effect sizes, i.e., containing weak, medium, and strong correlations. These were generated by defining *y* = *x* + *e*, where *x* are *n* samples drawn from a normal distribution with mean 0 and standard deviation 1, and *e* are data drawn from a normal distribution with mean 0 and standard deviation 1 (strong), 2 (medium), or 3 (weak). We then calculated the correlation between *x* and *y* for Pearson’s *r*, Spearman’s *rho*, skipped correlation *r*’, and Shepherd’s *pi* (see Appendix 2), and determined if it passed the threshold for statistical significance. Four sample sizes were tested: *n* = 10, 30, 60, and 100. The resulting proportions of significant tests reflect statistical power over 10,000 independent simulations.

### False positive simulations

To estimate the false positive rates for different tests we examined a range of statistical tests on simulated uncorrelated data. We simulated separate vectors *x* and *y* for three different data distributions: normal distribution, data containing a single outlier, and data containing bivariate outliers. Normal Gaussian data were generated by drawing *n* samples from a normal distribution with mean 0 and standard deviation 1. For data containing a single outlier we drew *n* − 1 samples from a normal distribution with mean 0 and standard deviation 1, and adding one observation drawn from a normal distribution with mean 0 and standard deviation 3. For data containing bivariate outliers we drew *n* − *m* samples from a normal distribution with mean 0 and standard deviation 1, and adding *m* observations drawn from a bivariate normal distribution with mean (0,0) and covariance matrix (3, 4.5; 4.5, 9). We defined *m* = *n*/20 rounded up (so *m* = 1 for *n* = 10, *m* = 2 for *n* = 30, *m* = 3 for *n* = 60, and *m* = 5 for *n* = 100). We then calculated the correlation between *x* and *y* for Pearson’s *r*, Spearman’s *rho*, skipped correlation *r*’, and Shepherd’s *pi* (see Appendix 2), and determined if it passed the threshold for statistical significance. Four sample sizes were tested: *n* = 10, 30, 60, and 100. The resulting proportions of significant tests reflect the false positive rate over 10,000 independent simulations. As can be seen in Figure 1B, bivariate outliers can drastically skew the outcome of Pearson’s *r* and Spearman’s *rho*; however, please note that in this case a small number of observations *are* in fact correlated because the outliers were drawn from a population of correlated data. We regard this as a test of spurious results because it simulates the situation where a correlation in some observations is due to artifactual reasons. This distinction would be harder to make in real observed data.

### Additional tests

In additional tests we also explored Kendall’s *tau* and the percentage bend correlation (Wilcox, 2005). The results for these tests are very comparable to Spearman’s *rho* and were therefore not included. Because the procedure for the minimum covariance estimator implemented in LIBRA is not identical to the procedure implemented by Wilcoxon *R*, we also ran additional tests of statistical power and false positive rates using the skipped correlation test (*scor*) in Wilcox’s R toolbox (both versions 14 and 16). Using the methods described by Rousselet and Pernet we edited the *scor* function to add the input argument MM = *T* where the *outpro* function is called, which means that the skipped correlation uses the median absolute deviation to the median to detect outliers. The results of these tests were comparable to the main results reported here.

## Appendix 2

### Shepherd’s PI correlation

The Mahalanobis distance (in squared units) measures the distance in multivariate space taking into account the covariance structure of the data. Because a few extreme outliers can skew the covariance estimate, *D*_m_ is not robust. We therefore bootstrap the Mahalanobis distance by resampling *n* observations with replacement (i.e., allowing duplicates) and then calculating the Mahalanobis distance for each *actual* observation from the bivariate mean of the resampled data. The bootstrapped Mahalanobis distance, *D*_s_, for each observation is the mean across the distances from all resamples. Normally we advise using 10,000 resamples; however, in the simulations shown here we used 1,000 resamples for the sake of expediency. In these analyses we used the MATLAB function *mahal* from the Statistics Toolbox to calculate Mahalanobis distances, but because this measure is clearly defined this can be achieved in other ways. Another free alternative is the *mahalanobis* function in the LIBRA toolbox.

Observations with *D*_s_ equal to or greater than six are then removed from the sample. We calculate Spearman’s *rho* over the remaining data. Shepherd’s *pi* is equal to *rho*. However, because removing data points can inflate false positive rates, the resulting *p*-statistic is then multiplied by two (naturally, with a cut-off of one) to account for outlier removal.

The MATLAB code for calculating Shepherd’s *pi* and for displaying scatter plots incorporating the bootstrapped Mahalanobis distance and denoting removed outliers as shown in Figure 1C and Figures A1 and A2 in Appendix can be downloaded from the author’s website (http://www.fil.ion.ucl.ac.uk/~sschwarz/Shepherd.zip).
